# New Mode of Preserving Bodies for Dissection

**Published:** 1846-06

**Authors:** 


*New Mode of Preserving Bodies for Dissection.*—Several years since a patent was obtained by the distinguished head of the medical department of the navy, Sir William Burnett, for a process discovered by him for preserving timber, canvass, &c., from dry rot, mildew, &c. The preserving substance is *chloride of zinc* in solution, and has been proved to be of perfect efficacy, by the most extensive and long-continued trials. Latterly the solution has been applied to the uses of the dissecting-room, and, as will appear from the following testimonials, with the most decided good effects.

14, *Golden Square, Feb.* 13, 1846.

My dear sir,—It affords me much pleasure to state the result of some experiments I have made with your patent fluid, with the view of testing its value as a preservative of animal structures prepared by the anatomist. This will not require many words. When used in a proper degree of dilution (about one part to fifty of water), its success is complete, and it appears to me to preserve the colour and texture of the parts very admirably. It has the further very important advantage, of not acting on the steel instruments employed, being, in this respect, equal to alcohol. I consider this fluid as a very valuable addition to the means hitherto at our command for such objects.

Believe me, my dear sir, ever faithfully yours,

W. Bowman, F. R. S.,
Demonstrator of Anatomy in King's College,
and Assist.-Sur. of King's Coll. Hosp.

To Sir W. Burnett, K. C. H. &c.

*University College, London, Oct.* 29, 1845.

My dear Sir William,—I gladly state the result of the trials I have hitherto made of the chloride of zinc, as a preservative of the dead body for anatomical purposes. * * *

The liquor not only prevents the access of putrefaction, but corrects its effects, and arrests its further progress when employed after the process has commenced. I witnessed a most signal instance of its efficacy in correcting offensive putrescence, and checking further decomposition, during the warm and moist weather of July, and can speak most confidently on the point.

We are now trying the qualities of the solution as a liquid for preserving wet preparations in jars, for which purpose I think it ought to answer well. I shall let you know the result of our trials when sufficiently advanced.

I remain, my dear Sir William, yours faithfully,

W. Sharpey.

To Sir W. Burnett, K. C. H. &c.

30, *Chester Street, March* 24, 1846.

Dear Sir William,—I have now used extensively your preparation for the preservation of animal matter, and find that it succeeds most completely.

From the 3d to the 28th February, I lectured upon the superior extremity. The arm, when received, was green in colour, and highly offensive. I injected the arteries with the fluid, mixed in the proportion of one pound of chloride of zinc to three gallons of water; and as the dissection proceeded, the surface of the limb was sponged about every alternate day with the solution. It was perfectly restored to its former fresh condition.

I have also placed various portions of the body in the solution, and they at present remain quite free from putrefaction. All our subjects are now injected with the fluid immediately they arrive; and I do not hesitate to say, that our dissection room is more free from unpleasant odour than any room of the kind in the metropolis; and the great advantage this fluid possesses over all others we have as yet tried is, that it has no effect upon the knives.

I cannot but consider it one of the greatest boons conferred upon the profession. Dissection may be carried on in the hottest weather without the slightest injury to the health, or offence from smell.

Believe me, dear Sir William, your very faithfully and obliged,

W. Veralius Pettigrew,
Lecturer upon Anatomy and Physiology at
St. George's School of Anatomy and Medicine.

To Sir Wm. Burnett, K. C. H. *Lon. Med. Gaz.*

